# Weak magnetic fields alter stem cell–mediated growth

**DOI:** 10.1126/sciadv.aau7201

**Published:** 2019-01-30

**Authors:** Alanna V. Van Huizen, Jacob M. Morton, Luke J. Kinsey, Donald G. Von Kannon, Marwa A. Saad, Taylor R. Birkholz, Jordan M. Czajka, Julian Cyrus, Frank S. Barnes, Wendy S. Beane

**Affiliations:** 1 Department of Biological Sciences, Western Michigan University, Kalamazoo, MI 49008, USA.; 2 Department of Electrical, Computer, and Energy Engineering, University of Colorado Boulder, Boulder, CO 80309, USA.

## Abstract

Biological systems are constantly exposed to electromagnetic fields (EMFs) in the form of natural geomagnetic fields and EMFs emitted from technology. While strong magnetic fields are known to change chemical reaction rates and free radical concentrations, the debate remains about whether static weak magnetic fields (WMFs; <1 mT) also produce biological effects. Using the planarian regeneration model, we show that WMFs altered stem cell proliferation and subsequent differentiation via changes in reactive oxygen species (ROS) accumulation and downstream heat shock protein 70 (Hsp70) expression. These data reveal that on the basis of field strength, WMF exposure can increase or decrease new tissue formation in vivo, suggesting WMFs as a potential therapeutic tool to manipulate mitotic activity.

## Teaser

Weak magnetic fields affect reactive oxygen species levels, stem cell proliferation/differentiation, and new tissue growth.

## INTRODUCTION

Exposure to electromagnetic fields (EMFs) occurs both from modern technology and Earth’s natural geomagnetic field, which averages 25 to 65 μT (*1*). In many circles, it is assumed that the quantum of energy associated with these weak magnetic fields (WMFs; <1 mT) is too insubstantial to be biologically important (*2*). Despite the fact that stronger magnetic fields are known to affect chemical reaction rates and free radical concentrations (*3*, *4*), initial studies of WMF effects on cell cultures produced contradictory results. While one study reported increased levels of the transcription factor c-Myc in human leukemia cells following WMF exposure, a different group failed to replicate these results (*5*, *6*). Another study showed that WMFs stimulated protein tyrosine kinases Lyn and Syk levels in B-lineage lymphoid cells, while two later studies found no significant differences (*7*–*9*). However, recent evidence indicates that WMFs can affect biological systems in multiple ways. WMF exposure increased intracellular calcium concentrations and the rate of cellular development in satellite cells, and caused embryo mortality as well as altered vertebrae development in roach embryos (*10*, *11*). Cell-dependent effects from WMFs were seen in rat renal versus cortical astrocyte cells, with decreased levels of apoptosis, proliferation, and necrosis in renal cells but increases in all three in astrocyte cells (*12*). WMFs were also found to produce transient induction of the membrane permeability transition and increased cytosolic cytochrome c levels in human amniotic cells via an increase in reactive oxygen species (ROS) (*13*).

A theoretical basis exists for the effects of WMFs on the concentration of free radicals such as ROS, as outlined in (*14*–*16*). Traditionally viewed as harmful, ROS can trigger cell death and thus are highly regulated by antioxidant enzymes such as superoxide dismutase (SOD), but ROS are also beneficial—acting as regulatory mediators (*17*), assisting in muscle repair (*18*), and modulating cell signaling (*19*). More recently, ROS signaling has been shown to regulate new tissue growth, as such in zebrafish where ROS production triggers apoptosis-induced compensatory proliferation required for regeneration (*20*).

In this study, we sought to determine whether WMFs could produce biological effects in vivo (in whole organisms) using the robust planarian regeneration model. Planaria are free-living flatworms that are capable of regenerating all tissues, including the central nervous system and brain, owing to a large adult stem cell (ASC) population that comprises ~25% of all cells (*21*). After injury, ASCs mount an animal-wide proliferative response that initially peaks at ~4 hours; this is followed by ASC migration to the wound site over the first 72 hours, when a second mitotic peak occurs (*22*). This activity produces the blastema, a collection of unpigmented ASC progeny that forms the core of new tissues. Full regeneration of missing structures occurs in 2 to 3 weeks through the combination of new tissue growth and the apoptotic remodeling and scaling of old tissues.

## Results and discussion

**Fig. 1:**
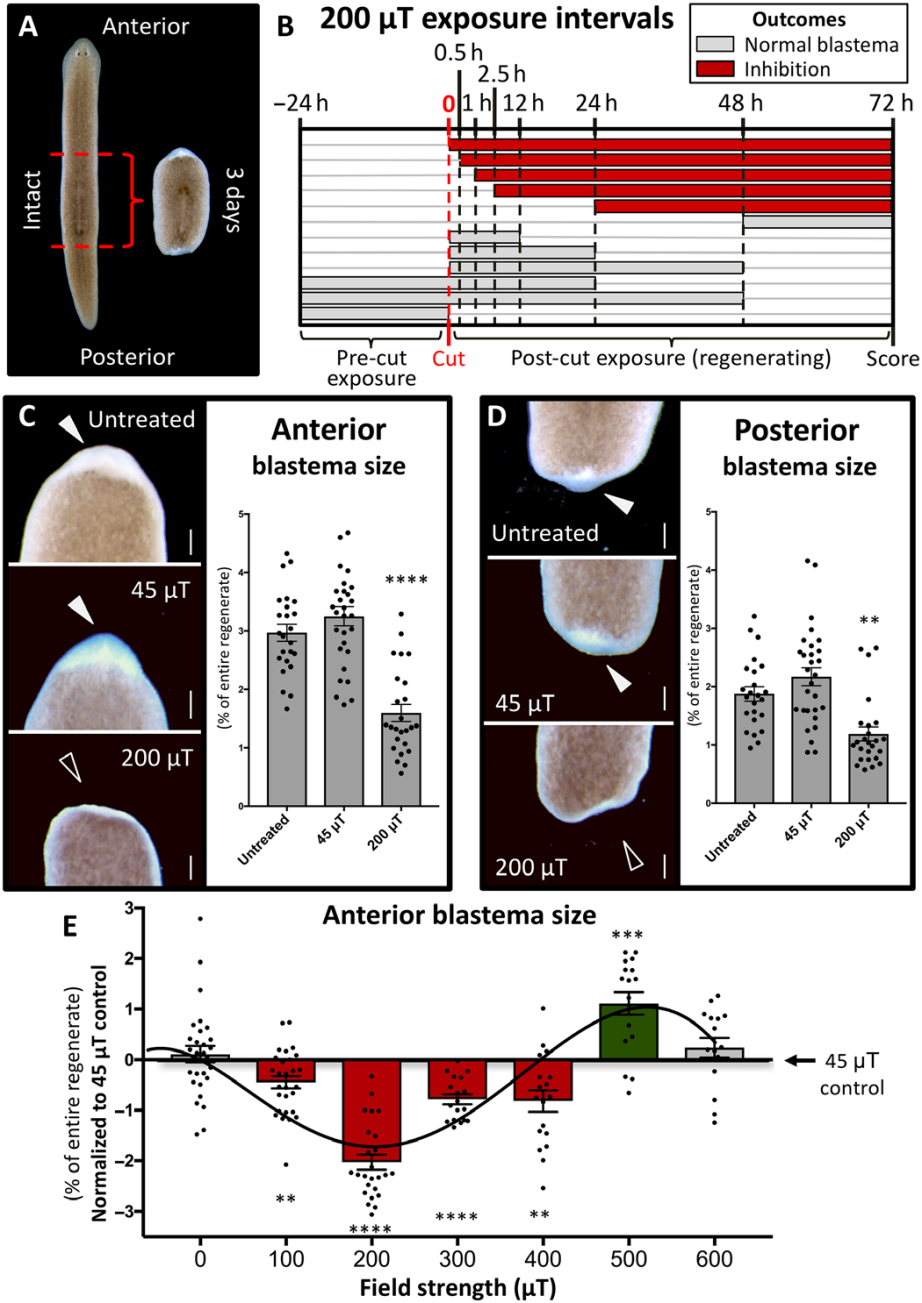
WMFs alter planarian regeneration. (**A**) Composite image illustrating *Schmidtea mediterranea* amputation scheme. (**B**) Temporal analyses of 200 μT WMF exposure on anterior blastema size. Each row represents an experimental group of pharynx fragments that were exposed at the indicated times and scored at 3 dpa. The length of each bar is the duration of 200 μT exposure. Red bars, blastema inhibition (Student’s *t* test against 45 μT; *P* ≤ 0.05). Gray bars, no effect. *n* ≥ 12 for all conditions. (**C** and **D**) Blastema size following 200 μT exposure versus untreated and 45 μT controls. Arrowheads indicate presence (solid) or lack (open) of blastema. Scale bars, 200 μm. One-way analysis of variance (ANOVA) with Tukey’s multiple comparison test; *n* ≥ 24. (**E**) Blastema size following exposure to different field strengths. Student’s *t* test against 45 μT; *n* ≥ 16. Red bars, reduced blastema size. Green bar, increased blastema size. Gray bars, no effect. For all: **P < 0.01, ***P < 0.001, and ****P < 0.0001; error bars are SEM; anterior is up; and animals scored at 3 dpa.

To determine whether WMFs affect tissue growth during planarian regeneration, we amputated animals above and below the pharynx (feeding tube) and examined blastema outgrowth at 3 days postamputation (dpa) (Fig. 1A) following WMF exposure. The setup of our magnetic field apparatus is outlined in fig. S1. We found that 200 μT WMF exposure produced blastema sizes that were significantly reduced as compared to both untreated and Earth-normal 45 μT field strength controls (Fig. 1, C and D). Temporal analyses, where regenerates were exposed for different lengths of time during the first 72 hours of regeneration (Fig. 1B), revealed that 200 μT exposure was required early and must be maintained throughout blastema formation to affect growth [24 hours postamputation (hpa) to 3 dpa]. Because shorter, single-day exposures failed to affect blastema size, these data suggest the presence of recovery mechanisms to ensure initiation of new growth. Furthermore, we found that WMFs produced field strength–dependent effects: Significant reductions of blastema size were observed from 100 to 400 μT, but conversely, a significant increase in outgrowth occurred at 500 μT (Fig. 1E).

**Fig. 2:**
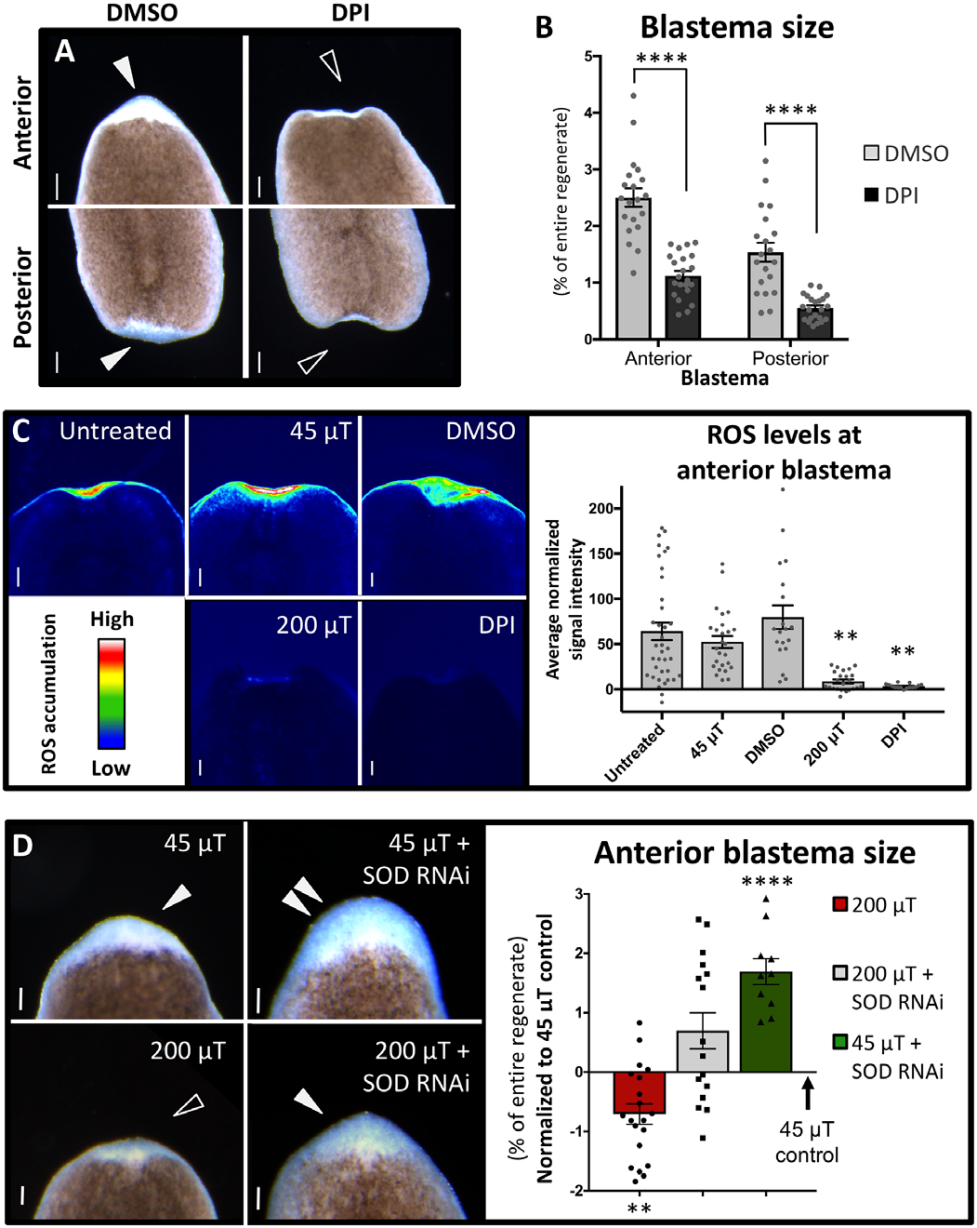
WMFs affect ROS levels during early regeneration. (**A** and **B**) Pharmacological ROS inhibition using 10 μM diphenyleneiodonium chloride (DPI) scored at 3 dpa. Student’s *t* test; *n* ≥ 20. Scale bars, 200 μm. DMSO, dimethyl sulfoxide. (**C**) Anterior ROS accumulation detection 1 hpa using the general oxidative stress indicator dye 5-(and-6)-chloromethyl-2′,7′-dicholorodihydrofluorescein diacetate (CM-H_2_DCFDA). One-way ANOVA with Tukey’s multiple comparison test; *n* ≥ 15. Scale bars, 200 μm. (**D**) RNAi of SOD imaged 3 dpa. Student’s *t* test against 45 μT; *n* ≥ 10. Scale bars, 100 μm. Red bar, reduced blastema size. Green bar, increased blastema size. Gray bar, no effect. For all: Solid arrowheads indicate normal blastemas; open arrowheads, lack of blastema; and double arrowheads, increased blastema; **P < 0.01 and ****P < 0.0001; error bars are SEM; and anterior is up.

We hypothesized that WMF effects were due to altered ROS levels, which peak at the wound site by 1 hpa and are required for planarian blastema formation (*23*). Pharmacological ROS inhibition resulted in significantly reduced blastema sizes (Fig. 2, A and B), phenocopying 200 μT WMF exposure. To determine whether WMF exposure altered ROS levels, we used a cell-permeant fluorescent general oxidative stress indicator dye to examine ROS accumulation during regeneration. Our results revealed that ROS levels were significantly reduced and/or absent from the wound site after both 200 μT exposure and direct ROS inhibition, as compared to controls at 1 hpa (Fig. 2C). Furthermore, increasing ROS levels via SOD inhibition by RNA interference (RNAi) was sufficient to completely rescue regenerative outgrowth in 200 μT–exposed regenerates (Fig. 1D and fig. S2, A and B). These data suggest that WMF effects on new tissue production are largely due to manipulation of ROS levels in vivo. We also found that SOD inhibition alone was sufficient to significantly increase blastema sizes in 45 μT controls (Fig. 2D), suggesting that tissue growth is highly dose dependent on ROS levels. This is supported by measurements at 500 μT, which also resulted in increased growth, that revealed increased ROS levels (average signal intensity of 61.7 for 500 μT versus 17.7 for 45 μT controls; *n* = 12; *P* < 0.01 by Student’s *t* test).

**Fig. 3:**
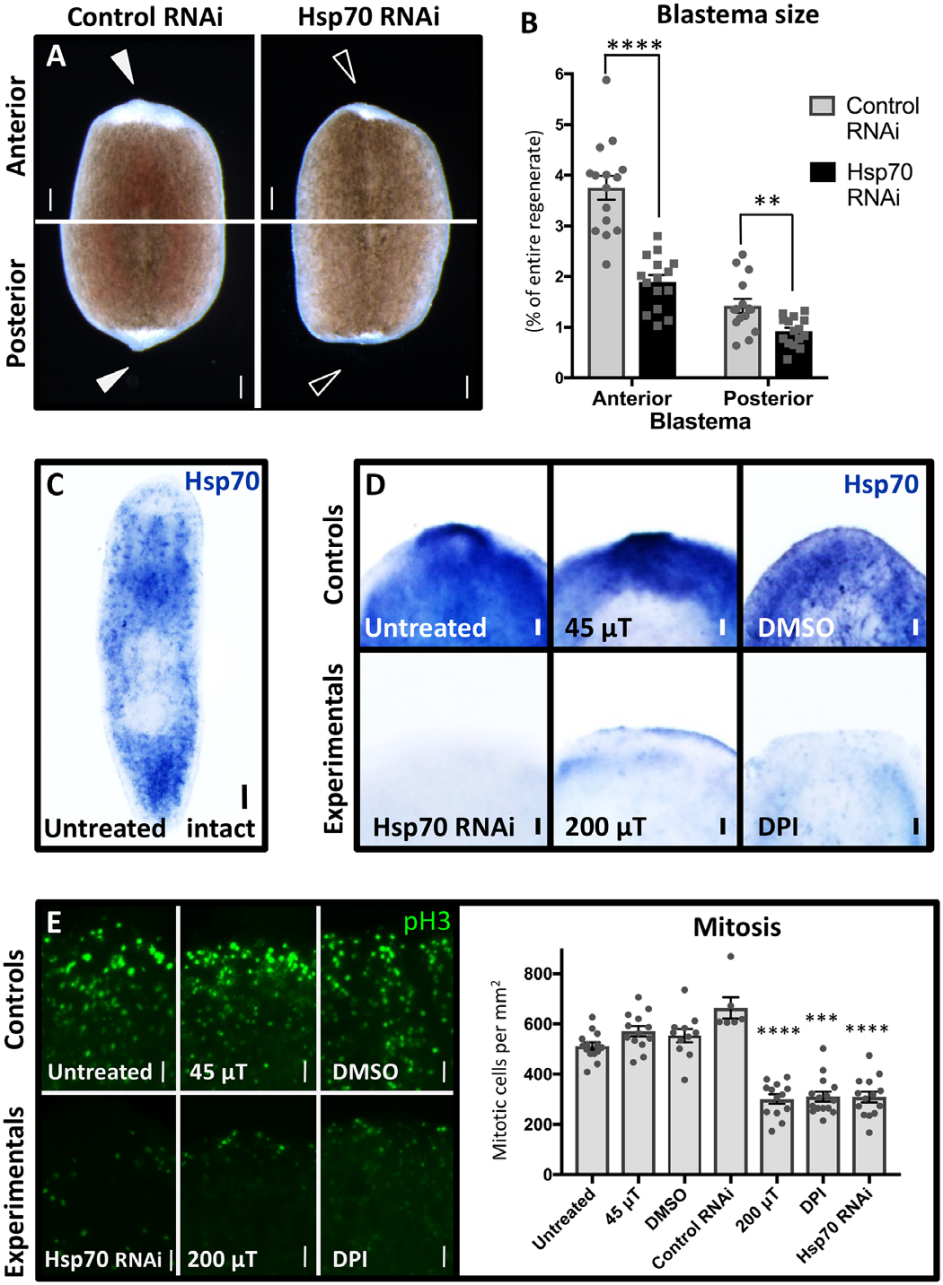
WMF effects on new tissue growth are caused by changes in both Hsp70 expression and proliferation. (**A** and **B**) Hsp70 RNAi scored at 3 dpa. Student’s *t* test; *n* ≥ 15. Arrowheads indicate presence (solid) or lack (open) of blastema. Control RNA: Venus-GFP. Scale bars, 200 μm. (**C**) Untreated intact animal whole-mount in situ hybridization (WISH) with the Hsp70 probe (*n* = 13). Scale bar, 200 μm. (**D**) Effects on Hsp70 expression visualized by WISH at 3 dpa. The anterior region is shown (*n* ≥ 5). Scale bars, 100 μm. (**E**) Phospho–histone H3 (pH3) staining of whole regenerates at 4 hpa. Only the anterior region is shown in the images. One-way ANOVA with Tukey’s multiple comparison test; *n* ≥ 6. Scale bars, 50 μm. For all: DPI used at 10 μM; **P < 0.01, ***P < 0.001, and ****P < 0.0001; error bars are SEM; and anterior is up.

To investigate genetic mechanisms by which ROS levels (and thus WMFs) regulate regenerative outgrowth, we examined their effects on heat shock protein 70 (Hsp70) expression. Hsp70 is a stress response protein that acts as a chaperone for protein folding during repair, promoting both normal cell survival and cancer cell growth (*24*). ROS have been shown to affect Hsp70 expression in cell culture (*25*), and cadmium exposure (which decreases SOD activity and thus increases ROS levels) alters expression of heat shock proteins in a dose-dependent manner (*26*). Our results demonstrate that Hsp70 inhibition by RNAi significantly reduced blastema sizes during planarian regeneration (Fig. 3, A and B), similar to 200 μT WMF exposure and direct ROS inhibition. Furthermore, Hsp70 expression was lost following both 200 μT exposure and direct ROS inhibition (Fig. 3, C and D). Consistent with these data, increasing ROS levels via SOD RNAi was sufficient to rescue Hsp70 expression in 200 μT–exposed regenerates (fig. S2C). These data suggest that increased ROS levels lead to increased Hsp70 expression during planarian regeneration and that WMFs can alter both processes in vivo.

**Fig. 4:**
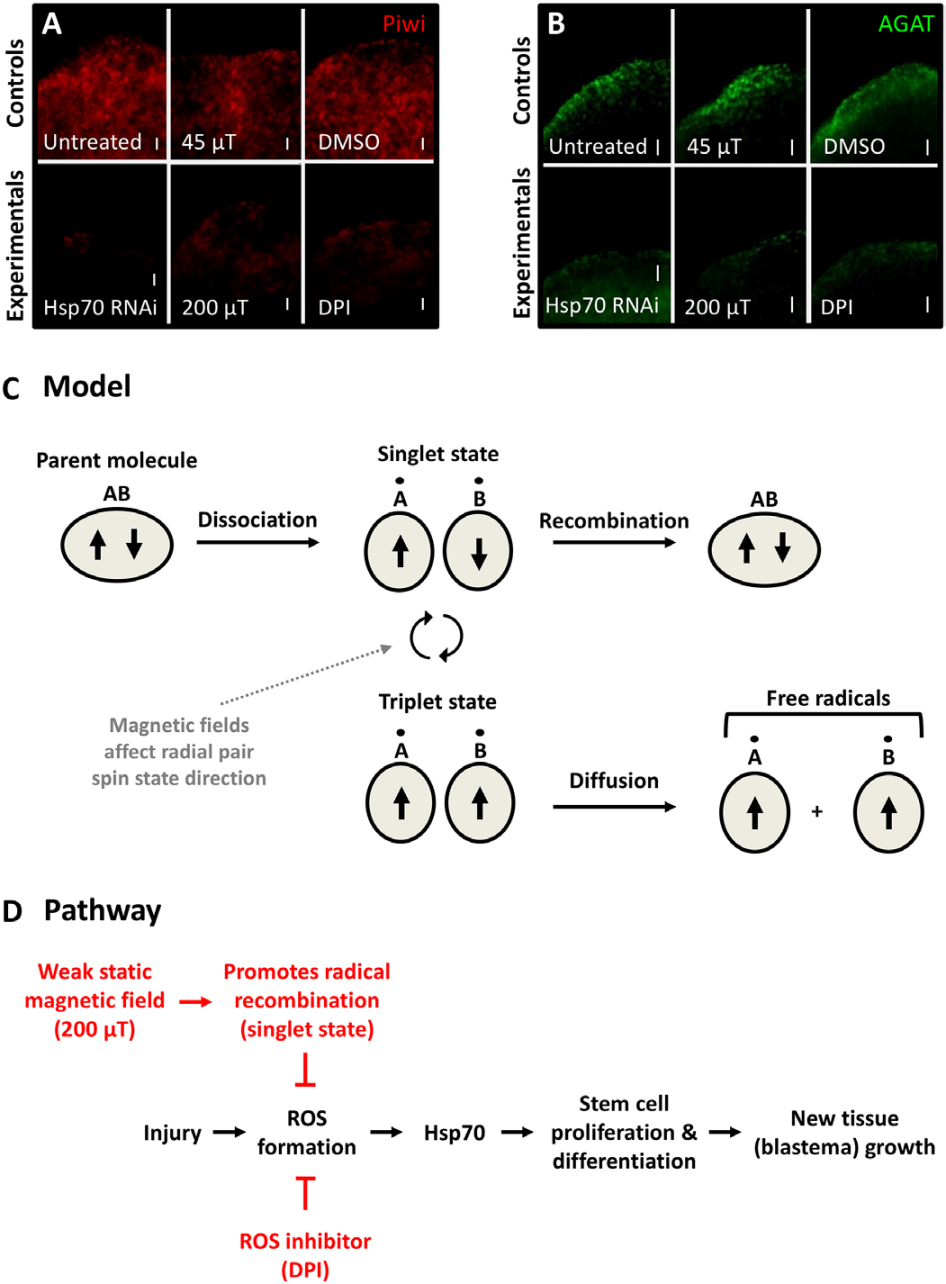
WMFs affect stem cell regulation during early regeneration. (**A** and **B**) Fluorescence in situ hybridization at 3 dpa to examine (A) the stem cell population (Piwi probe; *n* ≥ 6) and (B) stem cell differentiation (AGAT probe; *n* ≥ 5). The anterior region is shown. DPI used at 10 μM. Top panels are significantly different from bottom panels (Student’s *t* test; *P* ≤ 0.01). Scale bars, 50 μm. (**C**) Model for WMF effects on radical pair recombination. (**D**) Proposed pathway for 200 μT WMF effects on planarian regeneration.

To determine whether the observed changes in blastema size were due to changes in proliferation, we examined mitotic activity via phospho–histone H3 (pH3) staining at the wound site at 4 hpa. Our data revealed that 200 μT WMF exposure, direct ROS inhibition, and direct Hsp70 inhibition all resulted in significantly reduced mitotic activity as compared to control conditions (Fig. 3E). In planarians, ASCs are the only mitotically active cells, suggesting that WMFs (through ROS and Hsp70) affect stem cell activity. We used a planarian ASC marker (Piwi) to examine stem cell population levels during regeneration, as well as a late-progeny marker (AGAT) to examine stem cell differentiation. We found that 200 μT WMF exposure, direct ROS inhibition, and direct Hsp70 inhibition all resulted in significantly reduced ASC levels and stem cell differentiation near the blastema at 3 dpa (Fig. 4, A and B). Together, these data suggest that WMFs are able to alter stem cell regulation during regeneration via changes in ROS signaling.

Our data confirm that WMFs affect biological systems and establish a nascent mechanistic pathway by which this occurs. Currently, the main hypothesis for how magnetic fields interact with biological systems is through radical pair recombination (Fig. 4C) (*1*, *3*, *27*). In this model, components of a parent molecule can dissociate into a radical pair. Each unpaired electron will have opposing valence spin directions but may undergo a shift in spin direction. Antiparallel valence electron spins (singlet state) allow quick recombination of radicals back into the parent molecule. Alternatively, parallel spin states (triplet state) prevent recombination, providing sufficient time for the pair to diffuse away from one another, creating free radicals (*3*). Our data suggest that WMF exposure promotes singlet or triplet states depending on field strength, which results in decreased or increased ROS concentrations, respectively.

Our data reveal an underlying pathway by which WMFs affect planarian regeneration (Fig. 4D). WMF exposure alters ROS levels, which lead to changes in Hsp70 expression, which has consequences on stem cell proliferation and subsequent differentiation regulating blastema formation. It is likely that the effects on differentiation are the result of reduced numbers of proliferating stem cells, although direct effects cannot be ruled out. These findings are consistent with recent research highlighting the importance of ROS signaling in the cell in general (*13*, *17*–*19*) and in regeneration specifically (*20*, *23*, *28*, *29*). In addition, these data are consistent with studies that have linked EMF exposure to both increased Hsp70 expression and increased regeneration (*30*–*32*). Previous studies have also shown the importance of ROS signaling in initiating apoptotic-induced compensatory proliferation during regeneration (*20*). While our data demonstrate a link between WMFs and ROS-mediated stem cell proliferation, it is possible that effects on stem cell migration to the wound site and/or on apoptosis are also involved. Thus, future studies should investigate these mechanisms as possibilities for WMF effects on stem cell activity.

The ability of WMFs to modulate regenerative outgrowth in vivo suggests that WMFs could be a potential therapeutic tool. In support of this, our investigations with mouse fibroblast cells revealed that WMF exposure caused reduced growth of fibrosarcoma cell cultures but had no effect on noncancerous fibroblast controls (*33*). Together, these data suggest that highly proliferative cell populations may be specifically targeted during WMF exposure. If true, this would suggest novel possibilities for cancer treatments, where improved methods are needed to inhibit tumor growth while leaving surrounding cells unaffected.

## MATERIALS AND METHODS

### Animal care and amputations

An asexual clonal line of *Schmidtea mediterranea* (CIW4) was maintained at 18°C in the dark. All planarians were kept in worm water; worm water consists of Instant Ocean salts (0.5 g/liter) in ultrapure water of Type 1. Animals were fed no more than once a week with “natural” (no antibiotics or hormones) liver paste made from whole calf liver (Creekstone Farms). Liver was frozen and thawed only once before feeding animals. Worms 5 to 7 mm in length were starved at least 1 week before experimentation. *S. mediterranea* were amputated into trunk fragments via scalpel cuts made just above and below the pharynx. Amputations were done under a dissecting microscope on a custom-made cooling Peltier plate, as previously described (*34*). Untreated control animals were allowed to regenerate in standard biological oxygen demand incubators (VWR) at 18°C without light, which is the normal method for the planarian field.

### Magnetic field apparatus

A magnetic field enclosure (MagShield box) was constructed of μ-metal (which blocks magnetic fields) with a vertical μ-metal partition for running parallel experiments (control and experimental). Two custom-built triaxial Helmholtz coils were positioned in the exact center of each partition (via a stack of plastic well plates) to ensure that experiments were uniformly exposed to each specific magnetic field. Each coil was composed of a Plexiglas skeleton around which a ceramic-insulated copper wire was wrapped multiple times running in two parallel strands on each of the *x*, *y*, and *z* axes. Direct electric current to each coil was supplied by DC power sources (Mastech HY3005D-3) and was fed through the *x* and *y* coils in order to create a uniform static WMF. Before each experiment, Helmholtz coils were characterized using a Gauss meter (AlphaLab models GM1-ST or GM1-HS), which was also used to verify the magnetic field at the end of each experiment.

### Magnetic field exposure assay

The MagShield box was housed in a temperature-controlled room (20°C). The temperature inside the Helmholtz coils during magnetic field exposure assays was randomly tested twice a day over the 3-day assay, with an average temperature of 22°C (±1°C). Animals were placed in worm water in either 35 or 60 mm petri dishes at the indicated times into the MagShield box in the center of the partition (via a stack of larger plastic petri dishes). Animals were exposed to controlled static magnetic fields (in the dark) always in tandem: with one side of the MagShield partition set to Earth-normal 45 μT (for controls) and the other partition set to the indicated experimental WMF strength. Unless otherwise indicated, experimental planarians were exposed to 200 μT from 5 minutes postamputation (mpa) to 72 hpa. Experiments were repeated three times (except for untreated controls, which were performed once). Total biological replicates for each condition were as follows: untreated, *n* = 24; 45 μT, *n* = 29; and 200 μT, *n* = 25. For the temporal trials, planarians were exposed beginning at 0 hpa (i.e., 5 mpa), 30 mpa, 1 hpa, 2.5 hpa, 24 hpa, and 48 hpa until the end of the 72-hour period. In addition, planarians were exposed beginning at 0 hpa and ending at 12, 24, or 48 hpa, at which time they were removed from the MagShield box and allowed to continue regenerating in standard incubators (as for untreated controls) until the end of the 72-hour period. Last, planarians were preexposed starting 24 hours before amputation and were either allowed to regenerate with no further exposure or returned to the MagShield box to be further exposed for either 24 or 48 hpa (after which time they continued regenerating in standard incubators until the end of the 72-hour period). Temporal experiments were performed once (except for the 0- to 72-hour exposure, which was repeated three times, and the 30-min to 72-hour exposure, which was repeated twice). Total biological replicates for each condition were as follows: 0 to 72 hours, *n* = 25; 30 min to 72 hours, *n* = 19; 1 to 72 hours, *n* = 12; 2.5 to 72 hours, *n* = 14; 24 to 72 hours, *n* = 17; 48 to 72 hours, *n* = 16; 0 to 12 hours, *n* = 18; 0 to 24 hours, *n* = 20; 0 to 48 hours, *n* = 17; −24 to 24 hours, *n* = 13; −24 to 48 hours, *n* = 20; and −24 to 0 hours (amputation), *n* = 15. For the field strength trials, experiments were performed once, except for 200 μT, which was repeated three times, and 45 μT, which was repeated eight times (as every field strength was run with concurrent controls). Total biological replicates for each condition were as follows: 45 μT, *n* = 119; 0 μT, *n* = 30; 100 μT, *n* = 28; 200 μT, *n* = 25; 300 μT, *n* = 18; 400 μT, *n* = 18; 500 μT, *n* = 17; and 600 μT, *n* = 16.

### Pharmacological inhibition of ROS

ROS accumulation was inhibited with diphenyleneiodonium chloride (DPI; Sigma D2926). Animals were presoaked in 10 μM DPI [made from a 3 mM dimethyl sulfoxide (DMSO) stock] for 5 hours before amputation. Newly amputated pharynx fragments were immediately returned to 10 μM DPI and were allowed to regenerate until 72 hpa, at which time planarians were imaged for blastema size analyses. Control experiments were placed in DMSO (vehicle control). Total biological replicates for each condition were as follows: DMSO, *n* = 20 and DPI, *n* = 22.

### ROS indicator dye assay

The cell-permeant fluorescent general oxidative stress indicator dye, 5-(and-6)-chloromethyl-2′,7′-dicholorodihydrofluorescein diacetate (CM-H_2_DCFDA; Molecular Probes C6827), was used to visualize ROS accumulation (excitation, 470 nm; emission, 525 nm). One hour before imaging, worms were incubated in 25 μM CM-H_2_DCFDA made from 10 mM DMSO stock. For WMF experiments, following a 23-hour WMF preexposure period, whole worms were removed from the MagShield box, cut into pharynx fragments, placed into 25 μM CM-H_2_DCFDA, and returned to the MagShield box for the 1-hour incubation period. For ROS inhibition experiments, following a 5-hour pretreatment period, whole worms were removed from DPI, cut into pharynx fragments, and then placed into a combination of 10 μM DPI plus 25 μM CM-H_2_DCFDA for the 1-hour incubation period. After the 1-hour CM-H_2_DCFDA incubation period, all worms were rinsed three times in fresh worm water and then the ventral side was imaged using 35 mm FluoroDishes (WPI FD35-100) and 25 mm round no. 1.5 coverslips (WPI 503508). Signal intensity at the wound site was normalized to signal intensity of the central body to control for differences in dye loading between animals. Experiments were performed twice (except for DMSO and DPI). Total biological replicates for each condition were as follows: untreated, *n* = 37; 45 μT, *n* = 26; 200 μT, *n* = 24; DMSO, *n* = 19; and DPI, *n* = 15.

### RNA interference

RNAi was performed via feeding of in vitro–synthesized double-stranded RNAi, as previously described (*35*). A 489 base pair (bp) region of *S. mediterranea* SOD (SMU15011417) was used to generate SOD RNAi. The primers were 5′-ACTGGAGCCATCAATATCTGG and 3′-TAATCCGGCCTTACATTTTTG. A 552 bp region of *S. mediterranea* Hsp70 (SMU15039086) was used to generate Hsp70 RNAi. The region was from 5′-GGTTTTTGATTTGGGTGGTG to 3′-AGCTGTTGCTATGGGAGC. Worms were fed with RNAi three times over 8 days before being amputated on day 9, as indicated above. Control RNAi was double-stranded RNA to Venus-GFP, which is not present in the planarian genome. SOD RNAi rescue experiments were performed twice (except for 45 μT + SOD RNAi). Total biological replicates for each condition were as follows: 45 μT, *n* = 20; 200 μT, *n* = 20; 45 μT + SOD RNAi, *n* = 10; and 200 μT + SOD RNAi, *n* = 20. Total biological replicates for each condition for Hsp70 RNAi morphology experiments were as follows: control RNAi, *n* = 15 and Hsp70 RNAi, *n* = 15.

### Immunostaining and in situ hybridization

Immunostaining was performed as previously described (*34*). The primary antibody used was anti-pH3 (Sigma/Millipore 04-817; 1:25). The secondary antibody used was goat anti-rabbit horseradish peroxidase (Invitrogen 65-6120) with TSA Cyanine 3 (Cy3)–tyramide amplification (PerkinElmer; 1:50). Total biological replicates for each condition were as follows: untreated, *n* = 14; control RNAi, *n* = 6; Hsp70 RNAi, *n* = 14; 45 μT, *n* = 14; 200 μT, *n* = 13; DMSO, *n* = 12; and DPI, *n* = 15.

Colorimetric whole-mount in situ hybridization (WISH) was performed as previously described (*35*). A 601 bp region of *S. mediterranea* SOD was used to generate riboprobe. The region was from 5′-ACAACGGCAATGAACTTATTAATA to 3′-TAATCTTAATATTGCTCTTGAAC. Total biological replicates for each condition for the SOD probe were as follows: control RNAi, *n* = 13 and SOD RNAi, *n* = 10. Riboprobe for Hsp70 was generated from the same region as the RNAi. Total biological replicates for each condition for the Hsp70 probe were as follows: intact, *n* = 13; untreated, *n* = 13; Hsp70 RNAi, *n* = 12; 45 μT, *n* = 5; 200 μT, *n* = 5; DMSO, *n* = 5; DPI, *n* = 7; 200 μT + control RNAi, *n* = 10; and 200 μT + SOD RNAi, *n* = 8. Fluorescence in situ hybridization was carried out as previously described (*36*), with the following exceptions: Both prehybe and hybe used a yeast RNA concentration of 1 mg/ml, and probes were diluted to 0.5 ng/μl and hybridized for 24 hours. A 404 bp region of *S. mediterranea* AGAT-1 (NB.8.8b) was used to generate AGAT riboprobe. The primers were 5′-GGAGTTAAAGTGTCCATCCAG and 3′-GTTGCTAACCTGACTGACATGC. A 2461 bp region of *S. mediterranea* Piwi-1 (Q2Q5Y9.1) was used to generate the Piwi riboprobe. The region was from 5′-GATCCCAATTTAAGACCAAGAAGAG to 3′-TTTTTATGTATTCGATTAAAAAAAA. Total biological replicates for each condition for the Piwi probe were as follows: untreated, *n* = 7; Hsp70 RNAi, *n* = 9; 45 μT, *n* = 7; 200 μT, *n* = 7; DMSO, *n* = 6; and DPI, *n* = 6. Total biological replicates for each condition for the AGAT probe were as follows: untreated, *n* = 5; Hsp70 RNAi, *n* = 9; 45 μT, *n* = 6; 200 μT, *n* = 7; DMSO, *n* = 6; and DPI, *n* = 6.

### Image collection

Images were taken using a ZEISS V20 fluorescence stereomicroscope with AxioCam MRc or MRm camera and ZEN lite software (ZEISS). Regenerates were imaged while fully extended and moving to ensure the absence of any tissue bunching, which could affect analyses. Heat maps for visualizing intensity of ROS levels were generated using the standard rainbow lookup table (LUT) within the ZEN lite software. Adobe Photoshop was used to orient, scale, and improve clarity of images (but not for fluorescent images). Data were neither added nor subtracted; original images are available upon request.

### Quantification and statistical analyses

For blastema size, the magnetic lasso tool in Adobe Photoshop was used to generate total pixel counts for both the anterior and posterior blastemas (as well as the entire regenerate). To control for worms of different sizes, blastema sizes were expressed as a ratio of blastema size/total regenerate size. For ROS indicator dye assay, the magnetic lasso tool was used to obtain mean gray intensity values for both the anterior and posterior blastema, as well as baseline mean pixel intensity values (from the center of the regenerate). Signal intensity was expressed as average blastema pixel intensity − average baseline mean pixel intensity. For the pH3 assay, images were masked to avoid background signal and pH3^+^ cells were counted with the RTCN tool in ImageJ; the whole regenerate was measured with the magnetic lasso tool in Adobe Photoshop, and the final counts were shown as cells per square millimeter. Significance was determined using either a one-way analysis of variance (ANOVA) with Tukey’s multiple comparison test (using GraphPad Prism version 7.00 for Mac) or a two-tailed Student’s *t* test with unequal variance (using Microsoft Excel).

## Supplementary Material

http://advances.sciencemag.org/cgi/content/full/5/1/eaau7201/DC1

## SUPPLEMENTARY MATERIALS

Supplementary material for this article is available at http://advances.sciencemag.org/cgi/content/full/5/1/eaau7201/DC1 Fig. S1. Magnetic field enclosure (MagShield) setup. Fig. S2. Loss of SOD rescues 200 μT WMF exposure by increasing levels of ROS.
