# Supplementary material for: Weak magnetic fields alter stem cell–mediated growth
Source: Sci Adv. 2019 Jan 30;5(1):eaau7201. doi: 10.1126/sciadv.aau7201 (PMC6353618; doi:10.1126/sciadv.aau7201)
Supplement: http://advances.sciencemag.org/cgi/content/full/5/1/eaau7201/DC1 [file supp_5_1_eaau7201__index.html]

Science Advances | Science Advances

## Supplementary Materials

**This PDF file includes:**

- Fig. S1. Magnetic field enclosure (MagShield) setup.
- Fig. S2. Loss of SOD rescues 200 μT WMF exposure by increasing levels of ROS.

Download PDF

**Files in this Data Supplement:**

- Adobe PDF - aau7201\_SM.pdf
