# Supplementary material for: Weak magnetic fields alter stem cell–mediated growth
Source: Sci Adv. 2019 Jan 30;5(1):eaau7201. doi: 10.1126/sciadv.aau7201 (PMC6353618; doi:10.1126/sciadv.aau7201)
Supplement: http://advances.sciencemag.org/cgi/content/full/5/1/eaau7201/DC1 [file aau7201_SM.pdf]

Science Advances
AAAS

advances.sciencemag.org/cgi/content/full/5/1/eaau7201/DC1

Supplementary Materials for

Weak magnetic fields alter stem cell–mediated growth

Alanna V. Van Huizen, Jacob M. Morton, Luke J. Kinsey, Donald G. Von Kannon, Marwa A. Saad, Taylor R. Birkholz, Jordan M. Czajka, Julian Cyrus, Frank S. Barnes, Wendy S. Beane*

*Corresponding author. Email: wendy.beane@wmich.edu

Published 30 January 2019, *Sci. Adv.* **5**, eaau7201 (2019)
DOI: 10.1126/sciadv.aau7201

This PDF file includes:

Fig. S1. Magnetic field enclosure (MagShield) setup.
Fig. S2. Loss of SOD rescues 200 µT WMF exposure by increasing levels of ROS.

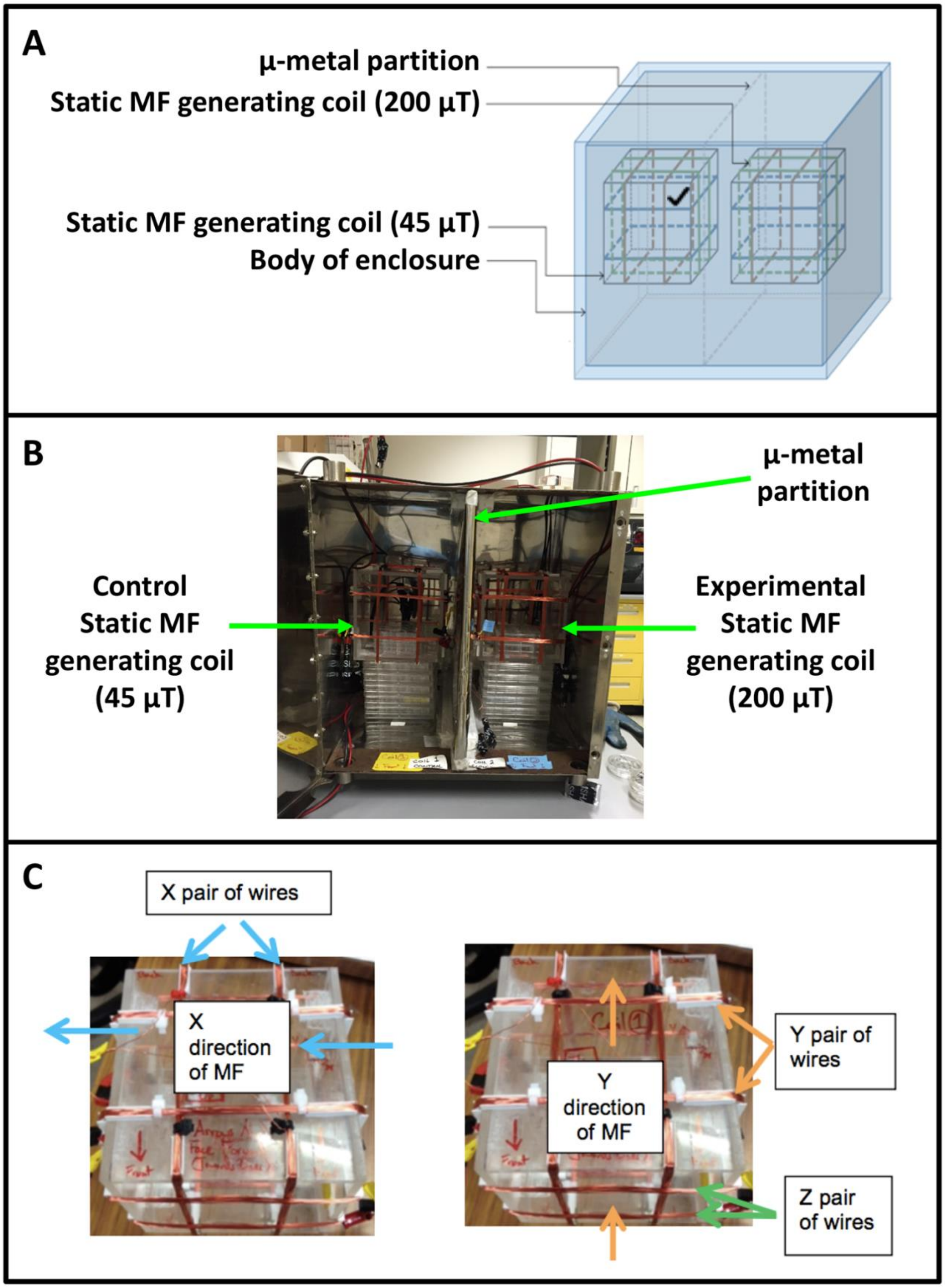

Fig. S1. Magnetic field enclosure (MagShield) setup. A) The MagShield box consists of a μ-metal enclosure with a vertical μ-metal partition separating control weak magnetic field (WMF) exposure (45 μT, indicated by checkmark on the left), from experimental conditions (right side). **B)** Photograph of the MagShield box. Each side has a stack of welled plates to position animals directly in the center of the generated WMF. **C)** Photos of the Helmholtz coils used to generate WMFs. Left side indicates position of "X" coils and the direction of WMF generated. Right side indicates position of "Y" coils and direction of WMF generated. Photo credit for B and C: Jacob M. Morton, Western Michigan University.

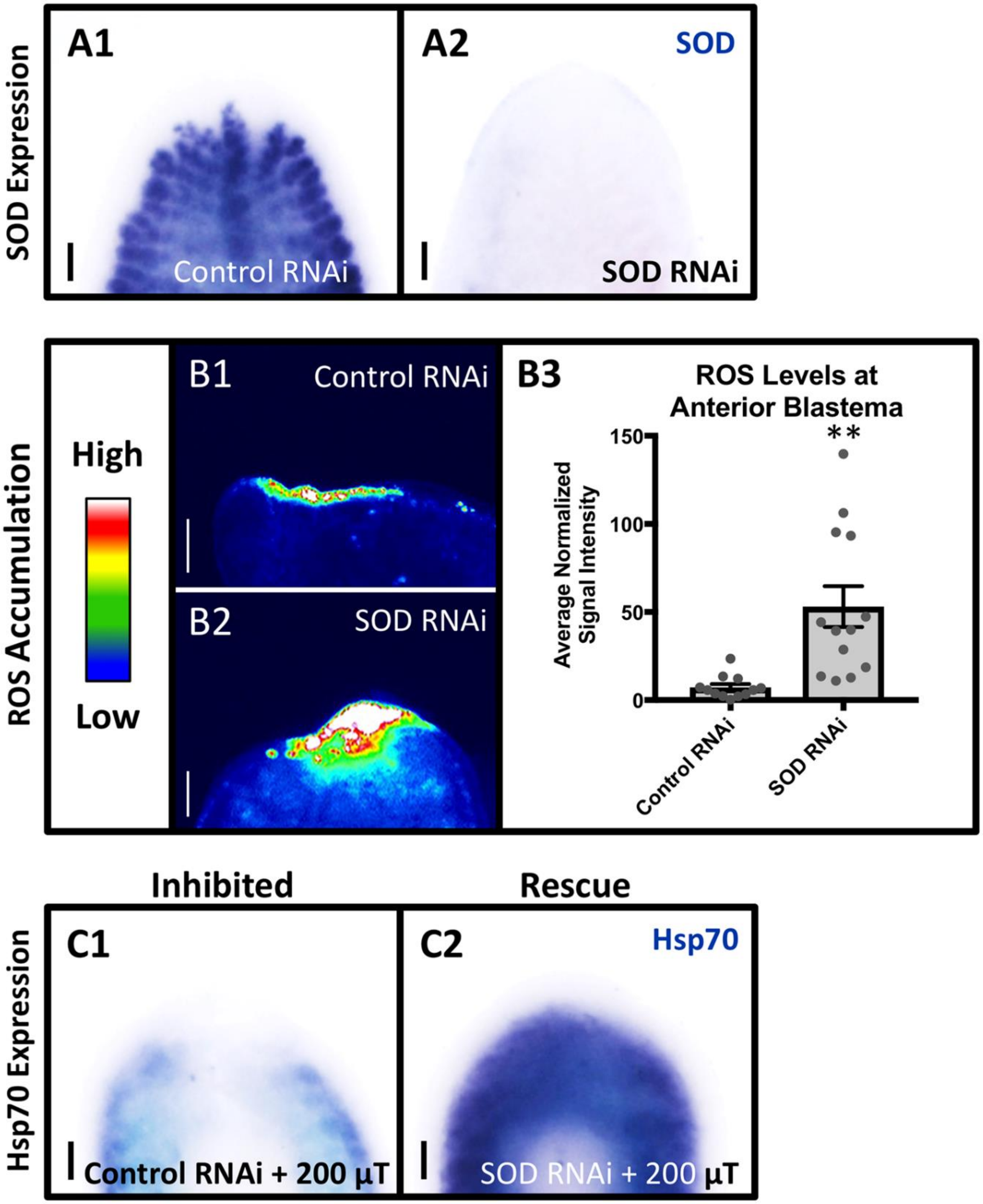

Fig. S2. Loss of SOD rescues 200 µT WMF exposure by increasing levels of ROS. A) Intact animal whole mount *in situ* hybridization (WISH) with SOD probe following (A1) Control RNAi (Venus-GFP) or (A2) SOD RNAi, showing that SOD RNA interference eliminates SOD expression. n ≥ 10. **B)** Anterior ROS accumulation detection 1 hour post amputation using the general oxidative stress indicator dye, CM-H_2DCFDA, following Control RNAi or SOD RNAi, showing that SOD RNAi results in increased ROS levels. **p<0.01 by Student's *t*-test compared to Control RNAi. n ≥ 12. **C)** Effects on Hsp70 expression visualized by WISH at 3 days post amputation following (C1) Control RNAi plus 200 µT WMF exposure or (C2) SOD RNAi plus 200 µT exposure, showing that SOD RNAi rescues Hsp70 expression. Penetrance of phenotypes shown: inhibited expression = 7/10; rescued expression = 6/8. For all: Anterior region shown, scale bars = 100 µm, error bars = SEM, and anterior is up.
